# Indigenous Youth and Resilience in Canada and the USA: a Scoping Review

**DOI:** 10.1007/s42844-022-00060-2

**Published:** 2022-05-23

**Authors:** Olivia Heid, Marria Khalid, Hailey Smith, Katherine Kim, Savannah Smith, Christine Wekerle, The Six Nations Youth Mental Wellness Committee, Tristan Bomberry, Lori Davis Hill, Daogyehneh Amy General, Tehota’kerá:tonh Jeremy Green, Chase Harris, Beverly Jacobs, Norma Jacobs, Katherine Kim, Makasa Looking Horse, Dawn Martin-Hill, Kahontiyoha Cynthia Denise McQueen, Tehahenteh Frank Miller, Noella Noronha, Savanah Smith, Kristen Thomasen, Christine Wekerle

**Affiliations:** 1 Health Sciences, Faculty of Health Science, McMaster University, Hamilton, Ontario Canada; 2 Life Sciences, Faculty of Science, University of Waterloo, Waterloo, Ontario Canada; 3 Social Work, School of Social Work, Dalhousie University, Halifax, Nova Scotia Canada; 4 Pediatrics, Offord Centre for Child Studies, McMaster University, Hamilton, Ontario Canada

**Keywords:** Indigenous youth, Resilience, Cultural continuity, Mental health

## Abstract

Relative to non-Indigenous youth, Indigenous youth have been under-represented when studying pathways to mental wellness. Yet, a broad range of adversity is acknowledged, from intergenerational and ongoing trauma arising from colonial policies. This scoping review explores resilience definitions, measures, key stressors, and what Indigenous youth identify as pathways to their wellness, based on quantitative and qualitative peer-reviewed literature in Canada and the Continental United States. Eight databases (*EBSCO*, *PsycINFO*, *Science Direct*, *Social Science Citation Index*, *Web of Science*, *PsycARTICLES*, and *EMBASE*) and hand searches of 7 relevant journals were conducted to ensure literature coverage. Two independent reviewers screened each article, with one Indigenous screener per article. The final scoping review analysis included 44 articles. In articles, no Indigenous term for resilience was found, but related concepts were identified (“walking a good path,” “good mind,” Grandfathers’ teachings on 7 values, decision-making for 7 generations into the future, etc.). Few Indigenous-specific measures of resilience exist, with studies relying on Western measures of psychological resilience. Qualitative approaches supporting youth-led resilience definitions yielded important insights. Youth stressors included the following: substance use, family instability, and loss of cultural identity. Youth resilience strategies included the following: having a future orientation, cultural pride, learning from the natural world, and interacting with community members (e.g., relationship with Elders, being in community and on the land). Indigenous traditional knowledge and cultural continuity serve as prominent pathways to Indigenous youth resilience. More research is needed to yield a holistic, youth-centered measure of resilience that includes traditional practices.

## Introduction

Indigenous people are defined by the World Health Organization (WHO) as “... populations or communities that live within, or are attached to, geographically distinct traditional habitats or ancestral territories, and who identify themselves as being part of a distinct cultural group, descended from groups present in the area before modern states were created and current borders defined. They generally maintain cultural and social identities, and social, economic, cultural, and political institutions, separate from the mainstream, or dominant society or culture” (WHO, n.d.). Every nation has unique cultural practices (e.g., coming-of-age ceremonies), ancestral lands, pre-contact and post-colonizer histories, and traditional stories (e.g., Creation story). Within the Canadian context, the government recognizes three distinct groups of Indigenous people: First Nations, Métis, and Inuit (Government of Canada, 2019). In the Continental United States, Indigenous peoples are recognized as constituting two broad groups: Native American peoples (AI) and Alaska Native (AN) peoples (Native American, n.d.). Native Americans can be further divided based on an area of residence, for example, Northern America (e.g., USA and Canada), resulting in certain tribes’ traditional lands spanning both the USA and Canada (e.g., Mohawk; Mohawk, n.d.).

In the USA, there are 574 tribes recognized, across 35 US states, with 9.7 million identifying as AI/AN (Foxworth et al., 2021). In Canada, over 1.6 million individuals identified as Indigenous in the 2016 census (Government of Canada, 2019). Indigenous populations are the single fastest-growing population and much younger on average than non-Indigenous Canadians (Government of Canada, 2018). Indigenous youth face a wide range of stressors such as suicidality, substance use, racism, discrimination, lateral violence, systemic violence, and family violence compared to non-Indigenous youth (Fitzgerald et al., 2017). Past colonial policies driving family, community, language, and cultural disruption are evident through population containment actions (e.g., introduction of novel viruses, forced attendance in residential “schools,” and over-representation in child welfare systems; Government of Canada, 2015; United Nations, 2015), and their related impacts. For example, in 2021, there were 58 long-term boil water advisories in 38 Indigenous communities (Indigenous Services Canada, 2021); further to this, many reservation communities experience unsafe housing, unsafe water, a lack of a sanitation system, and other ecological issues (e.g., encroachment and destruction of lands and their resources by corporations; United Nations, 2009). High rates of adverse childhood experiences (ACEs) and decreased well-being in Indigenous youth are commonly seen (Ames et al., 2015; Fitzgerald et al., 2017; BigFoot et al., 2018; Freeman & Ammerman, 2021; Richards et al., 2021; Smith et al., 2021). Renewed trauma burden exists with the current initiatives in uncovering residential school burial grounds. Despite the multi-level adverse contexts, Indigenous youth remain resilient, reflecting their resistance, persistence, and ability to thrive. However, to date, no systematic review of the literature has occurred from a resilience lens.

Resilience has numerous definitions. In Western science, resilience has evolved to denote ever-increasing complexity. Currently, resilience reflects processes (e.g., emotion regulation), promotive factors (e.g., available and “harnessed” instrumental and psychological resources), outcomes (e.g., academic achievement, quality of life), trajectories (e.g., stable healthy functioning), and the potentiality or capacity of a dynamic system to adapt (Southwick et al., 2014). These various aspects can be organized along Bronfenbrenner’s ecological model for “where” these resilience facets mainly exist (e.g., macro-, meso-, and micro-system levels; Ungar and Liebenberg, 2013). For this paper, we were guided by the broad definition provided by United Nations International Children’s Emergency Fund (UNICEF), “Resilience is understood by UNICEF as the ability of children, households, communities, and systems to anticipate, prevent, withstand, manage, and overcome cumulative stresses and shocks in ways which advance the rights of every child, with special attention to the most vulnerable and disadvantaged children. Supporting resilient development, therefore, means promoting risk-informed programming which includes development of nationally led common risk assessments, Disaster Risk Reduction, climate change adaptation, conflict prevention, and peacebuilding. UNICEF and partners can build resilience and reduce vulnerability by supporting the capacities of local systems and structures to address these systematically by integrating risk factors such as climate change into public services planning and delivery” (UNICEF, n.d.).

While Indigenous cultures focus on responsibilities as people of the land, Western approaches focus on rights. Given prior laws in various countries that prohibited cultural practice, the United Nations (UN) created the Declaration on the Rights of Indigenous People (UNDRIP) in 2007. This global recognition established Indigenous peoples’ *right* to identify as their own distinct group, practice traditional medicine, and have self-determination of their collective and individual well-being (United Nations, 2015). Practicing culture is recognized as a fundamental right and fundamental pathway to wellness that has been practiced prior to the establishment of countries. Building upon the efforts of the UNDRIP, some countries have established tribunals and commissions to address the recognition of the rights of Indigenous people, redress structural, and system rights infringement, and create an action plan for respectful relationships. In Canada, the Truth and Reconciliation Commission published their final report concluding that Indigenous people have long faced discrimination and cultural genocide in Canada, with 94 calls to action to effect positive change (Canada, 2015). The Truth and Reconciliation Commission has recognized that long-held colonial policies have had the effect of limiting the right to practice culture (e.g., ceremony) and learn culture (e.g., place-based knowledge, Native language, medicinal plants), necessary to pass along traditional ecological knowledge for ancestral holistic health, as well as the basis for Indigenous resilience.

Among the Indigenous languages with which we are familiar, there is no word for resilience per se. For example, among the Haudenosaunee, words are poly-synthetic and the morphemes string together to provide a complete thought. For example, among the Haudenosaunee, people of the longhouse, the Thanksgiving address is the center of their traditional way of life. The Mohawk word for giving thanks, *Tayethinonhwerá:ton*, breaks down into resilience components of who, what, how, where, and when. This word reflects a person who has balance with all entities, those that are seen (visible Earth) or unseen (spirit and sky world). This is represented by the dome shape of the world, reflected in the shape of the top of our heads, with the presence of the four directions (North, South, East, West; Wekerle & Boles, 2021). This level of consciousness is unlimited and heartfelt. The final section of the word denotes speaking it aloud into existence, such that all minds have come together to rejuvenate the community—feeling, sensing, and responding, as fast as the thought travels (Wekerle & Boles, 2021). Given the variation across Indigenous nations, a pan-Indigenous model of resilience, therefore, is not attainable.

A prior scoping review article on resilience in Indigenous youth found that either Western resilience definitions alone were used, or these were primarily combined with Indigenous concepts within a “two-eyed seeing” approach (Toombs et al., 2016). Given the scarcity of published studies, the current review, as well as the Toombs et al. (2016) review, included gray literature. However, the Indigenous youth resilience field has evolved to be able to conduct a scoping review of the peer-reviewed only literature, and focus more broadly on different Indigenous populations (i.e., beyond Canadian youth). The Toombs et al. (2016) study found that a key Indigenous youth resilience process was community connectedness. This new review strives to explore resilience in terms of study-specified definitions and measures, in addition to identifying youth resilience strategies leading to increased well-being. This review expands on the literature by looking at populations in both Canada and the Continental United States, providing a more holistic approach to determining resilience strategies among Indigenous youth. Additionally, this review aims to provide an update on the literature surrounding Indigenous youth resilience, since the publication of Toombs et al., in 2016.

## Methods

### Data Sources

A search of relevant databases and select individual journals was conducted to identify peer-reviewed literature published between January 1, 2008, and November 30, 2020. The year 2008 was selected as it includes articles post the significant UNDRIP document creation and adoption by the USA (Canada did not adopt it until 2016; Duncanson et al., 2021). This document supported recognition of Indigenous sovereignty, and the need for investment in Indigenous knowledge; hence, the authors hypothesize that the most relevant literature will be published in 2008 or later. Table 1 displays the databases and journals used in this study. Each database had an individually identified search strategy, developed in accordance with the subject headings used in the specific database. This review was guided by the input of a Six Nations of the Grand River–based community research committee composed of health researchers, health practice leaders, youth educators, and Elders who met regularly with the Indigenous youth lead author (OH). The search strategy was developed with the aid of an academic librarian and included terms related to Indigenous populations, adolescents, resilience, gender, and all states and provinces (see Appendix for a full list of terms). Gender was included as an effort to capture research involving two-spirit youth, an umbrella term reflecting a third gender and traditional role, where a male understands the spirit of a female, and vice versa, and not necessarily defining LGBTQ status (Government of Canada, 2020).

**Table 1:** Definitions of resilience at the ecological level of social interaction

Studies	Level of individual/community involvement and perspective used
Ames et al., 2015; Clark et al., 2013; Fraser et al., 2015; Gray et al., 2016; Harder et al., 2015; Kenyon & Carter, 2011; Krieg, 2016; Morton et al., 2020; Pertucka et al., 2016; Ranahan and Yuen, 2017; Snowshoe et al., 2017; Strickland and Cooper, 2011; Tiessen et al., 2009; Trout et al., 2018;	No explicit definition of resilience, instead used a broad concept of resilience that incorporates aspects of macro-/meso-levels of resilience from Western and Indigenous ideologies.
Gray et al., 2019; Hatala et al., 2020; Njeze et al., 2020; Ruttan et al., 2008; Ritchie et al., 2015; Sam et al., 2015; Sasakamoose et al., 2016; Stumblingbear-Riddle, 2012; Ungar et al., 2008; Wexler et al., 2013; Yeh et al., 2015	Macro-system Westernized definition of resilience
Barnett et al., 2020; Hatala et al., 2019; Mohatt et al., 2011; Sasakamoose et al., 2016; Ungar et al., 2008; Wexler et al., 2014, 2016; Isaacson, 2018; Kral et al., 2014; Sasakamoose et al., 2016; Freeman, 2017; Goodkind et al., 2012; Isaacson, 2018; Kral et al., 2014; Mohatt et al., 2011; Ruttan et al., 2008; Rasmus et al., 2014; Wood et al., 2018	Macro-system Indigenous definition of resilience
Baldwin et al., 2011; Barnett et al., 2020; Gray et al., 2019; Hatala et al., 2017, 2019; Isaacson, 2018; Kral et al., 2014; McMahon et al., 2013; Mohatt et al., 2011; Wexler et al., 2014	Meso-system Indigenous definition of resilience
Bruner et al., 2019; Kral et al., 2014; Victor et al., 2016	Micro-system Indigenous definition of resilience
Fitzgerald et al., 2017; Hatala et al., 2017; Ulturgasheva et al., 2014	Micro-system Westernized definition of resilience

In identifying search terms, a great deal of heterogeneity was found in the terms used to describe our population of interest—Indigenous youth. Youth was defined as spanning the adolescent to young adult years of 15 to 24 (i.e., United Nations Youth, n.d.). Over the years, various terms have been used to describe Indigenous peoples and the youth. To ensure that the most literature available was captured, terms that may now be considered politically incorrect or antiquated were used (e.g., Indian, Native, or Eskimo). The databases and journals included in our literature search are listed below.

Databases:

- EBSCO
- PsycINFO
- ScienceDirect
- Social Science Citation Index
- Web of Science
- PsycARTICLES
- Embase

Hand-searched journals:

- *Pimatisiwin*
- *Journal of Indigenous Wellbeing*
- *AlterNative: An international journal of Indigenous Peoples*
- *International Journal of Indigenous Health*
- *Canadian Journal of Native Studies*
- *International Indigenous Policy Journal*
- *Journal of Aboriginal Health*

Journals were hand searched to locate relevant studies that have possibly been inaccurately indexed or are not indexed at all, ensuring that relevant studies are not neglected. Additionally, it allows for studies that may not have been captured by the larger database searches to be incorporated into this review.

### Study Selection

Peer-reviewed articles written and published in 2008 or later were included in the analysis of this review. As UNDRIP was adopted in late 2007, our research group posited that an increased number of studies on the rights and well-being of Indigenous people would be published after the adoption of the declaration. As such, studies from 2008 to 2020 were included. Studies published previous to 2008 were not included in this review. Additionally, included articles focused on Indigenous populations in Canada, Continental United States (US), Hawaii, and Alaska. Studies on Indigenous populations outside of the Continental US including those in the US Affiliated Pacific Islands were excluded from the study. Eligible studies reported on youth in our age definition of 15–24 years of age. Studies including participants that were older or younger than the specified age range were excluded. In terms of resilience, studies had to include protective factors or strategies specific to Indigenous youth and communities. Studies were also included if they identified risk factors particular to Indigenous youth, although most also included resilience factors. Risk factors and resilience factors had to be reported directly from youth to be included in this review. Studies that had parents/guardians, other adults, or proxies reporting on resilience factors and strategies on behalf of youth were excluded from the analysis. All included studies were from published, peer-reviewed journals.

#### Data Extraction

The population type and location, sample size, research design, definition of resilience, resilience measures used, challenges to resilience, resilience factors, and resilience strategies used were extracted from each study and listed in Table 3. Not all studies provided data points for each category of interest. Comparisons between studies were drawn; however, by noting the specific location and community, we were cautious about generalizing about Indigenous peoples.

**Fig. 1:**
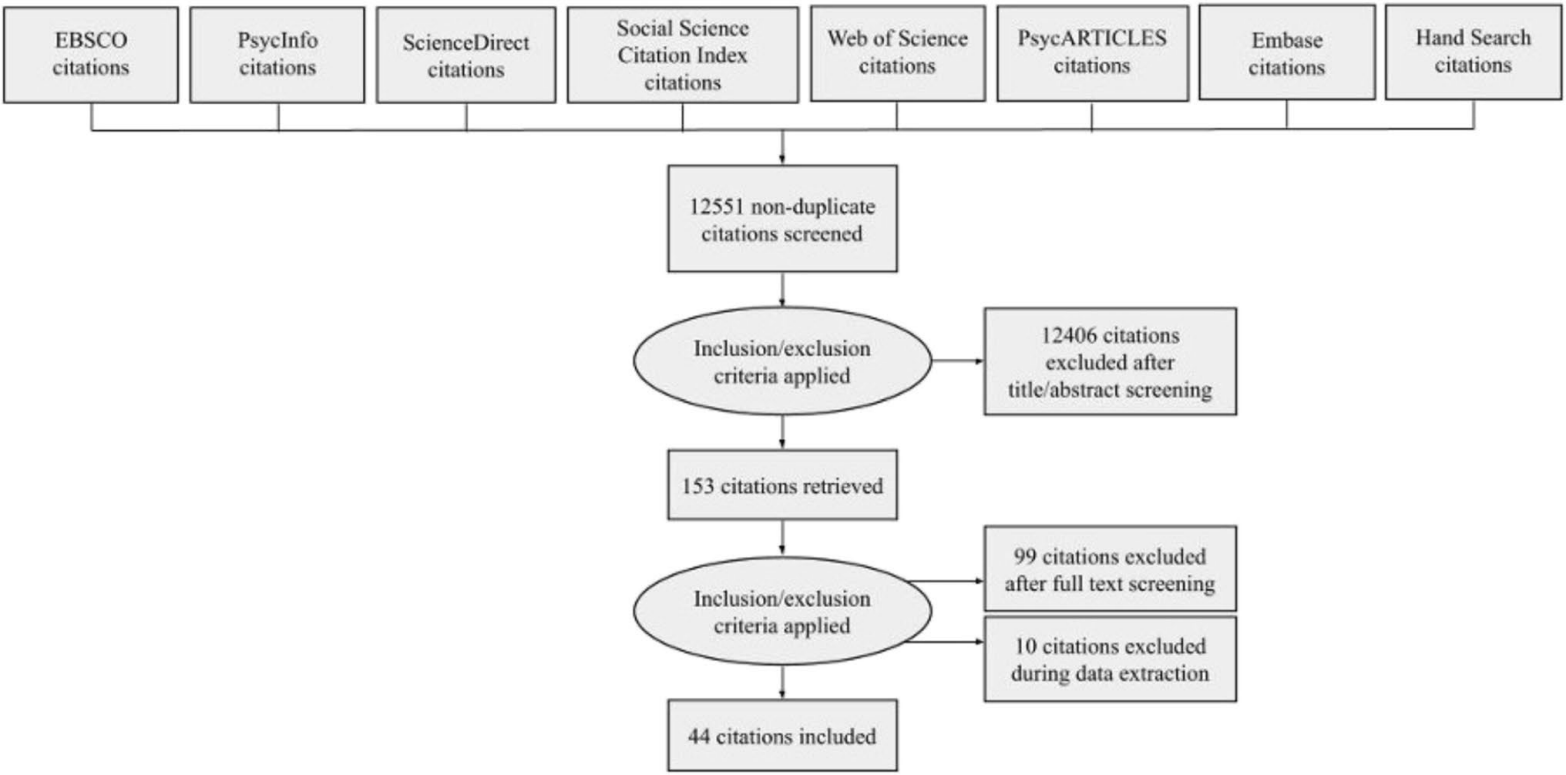
Literature search PRISMA diagram

Each study underwent several rounds of screening and review before inclusion in this scoping review. Studies identified by our search strategy were first screened by title and abstract screening, followed by a full-text review (Figure 1). During both stages of screening, each article was reviewed by two independent authors for inclusion. Two independent reviewers extracted relevant information from eligible studies according to our pre-specified extraction factors mentioned in Table 3. The methods used in conducting this scoping review ensure its accuracy and consensus through the use of inter-rater reliability, where at least two raters had to agree on the inclusion of a study. Additionally, to ensure results were not skewed, one reviewer of each paper was a member of an Indigenous community in Canada.

## Results

A summary of the results extracted from each paper can be found in Table 3. Of the 44 studies included in this review, 35 were from the databases searched and nine were from the hand searched journals. More than 50 Indigenous communities were included in this review. The studies selected for inclusion from the literature had a variety of different methods used to collect data. There were 26 that followed a primarily qualitative research design (Bruner et al., 2019; Fraser et al., 2015; Freeman, 2017; Goodkind et al., 2012; Hatala et al., 2017, 2019, 2020; Isaacson, 2018; Kral et al., 2014; Krieg, 2016; McMahon et al., 2013; Morton et al., 2020; Njeze et al., 2020; Ranahan & Yuen, 2017; Rasmus et al., 2014; Sasakamoose et al., 2016; Strickland & Cooper, 2011; Trout et al., 2018; Ulturgasheva et al., 2014; Ungar et al., 2008; Victor et al., 2016; Wexler, 2014; Wexler et al., 2013, 2014, 2016; Wood et al., 2018); 8 followed a quantitative design (Ames et al, 2015; Baldwin et al., 2011; Barnett et al., 2020; Fitzgerald et al. 2017; Gray et al., 2016; Kenyon & Carter, 2011; Mohatt et al. 2011; Snowshoe et al., 2017); and 10 were mixed methods studies (Clark et al., 2013; Gray et al., 2019; Harder et al., 2015; Ruttan et al., 2008; Pertucka et al., 2016; Ritchie et al., 2015; Sam et al., 2015; Stumblingbear-Riddle, 2012; Tiessen et al., 2009; Yeh et al., 2015). Community-consulted focus groups that used open-ended, semi-structured interviews were the most commonly used in qualitative and mixed-methods studies. This format provided a culturally based way to consider resilience, consistent with the oral and story-telling traditions among Indigenous peoples.

### Measures of Resilience

Quantitative studies focused primarily on the outcomes of mental health (particularly depressive symptoms, suicidal ideations), and protective factors measured at a single time point (Ames et al., 2015; Barnett et al., 2020; Gray et al., 2016, 2019; Kenyon & Carter, 2011). Mental health was measured using validated questionnaires, primarily Centre for Epidemiologic Studies Depression Scale (Ames et al., 2015; Baldwin et al., 2011), WHO Survey of Health Behaviours in School-Aged Children (Ames et al., 2015), General-Self Scale of the March Self-Description Questionnaire (Ames et al., 2015), General Mattering Scale (Barnett et al., 2020), and Positive and Negative Affect Schedule (Barnett et al., 2020; Kenyon & Carter, 2011; Tiessen et al., 2009). In terms of promotive or protective factors, many studies used the Rosenberg self-esteem scale (Barnett et al., 2020; Gray et al., 2016; Harder et al., 2015; Tiessen et al., 2009). Other questionnaires used to measure resilience and sense of community included the Child and Youth Resilience Measure, Child Version [CYRM-28; Isaacson, 2018], the Awareness of Connectedness Scale (ACS; Mohatt et al., 2011), Reasons for Life (RFL; Mohatt et al., 2011), and the Satisfaction with Life Scale for Children (Snowshoe et al., 2017).

Qualitative and mixed methods primarily used interviews, focus groups, and picture taking to allow youth to discuss individual and community strengths and relationships (Bruner et al., 2019; Fraser et al., 2015; Goodkind et al., 2012; Hatala et al., 2017, 2019, 2020; Kral et al., 2014; Krieg, 2016; McMahon et al., 2013; Morton et al., 2020; Njeze et al., 2020; Pertucka et al., 2016; Ranahan and Yuen, 2017; Rasmus et al. 2014; Sasakamoose et al., 2016; Strickland and Cooper, 2011; Trout et al., 2018; Ulturgasheva et al., 2014; Ungar et al., 2008; Victor et al., 2016; Wexler, 2014; Wexler et al., 2013, 2014, 2016; Wood et al., 2018). Mixed-methods studies also measured resilience quantitatively (Clark et al., 2013; Harder et al. 2015; Ritchie et al., 2015; Sam et al., 2015; Stumblingbear-Riddle, 2012; Yeh et al., 2015).

Studies reported individual quotes or images from youth to illustrate their thematic results (Clark et al., 2013; Hatala et al., 2017, 2019; McMahon et al., 2013; Mohatt et al., 2011; Wexler et al., 2014). Quotes provided indications of several resilience factors: “[referring to Elders] They are just like parents, but they are your grandparents . . . sometimes I can connect better with them because they’re not as strict as my parents. I have a great relationship with my grandparents. I see them . . . every weekend. Meals together are important. They are hilarious.” (Clark et al., 2013, p. 47). This quote not only identified intergenerational connection, but also the qualities of openness, acceptance, humor, and shared attention in the relationship. Intergenerational relationships as carriers of land-based identity were noted:

“It’s the same river that flows in my reserve that flows through the city here … my own reserve land and my grandma’s land is also here and it means a lot to me, because I know it. If I go down the river or I head up, where it takes me up north and it goes to my grandmother’s land, like either way, this is the road for me and I know this river, this water, and this place.” (Hatala et al., 2019, p. 125).

### Definition of Resilience, Resilience Factors, and Resilience Strategies

Of the 44 studies included, 14 did not provide an explicit definition of resilience, these studies used a general concept of resilience (Ames et al., 2015; Clark et al., 2013; Fraser et al., 2015; Gray et al., 2016; Harder et al., 2015; Kenyon & Carter, 2011; Krieg, 2016; Morton et al., 2020; Pertucka et al., 2016; Ranahan and Yuen, 2017; Snowshoe et al., 2017; Strickland and Cooper, 2011; Tiessen et al., 2009; Trout et al., 2018). In many studies, resilience was defined along Western conceptualizations, reflecting adaptive coping and persistence strategies (Gray et al., 2019; Hatala et al., 2020; Njeze et al., 2020; Ruttan et al., 2008; Ritchie et al., 2015; Sam et al., 2015; Sasakamoose et al., 2016; Stumblingbear-Riddle, 2012; Ungar et al., 2008; Wexler, 2014; Yeh et al., 2015). Other Western resilience concepts included the following: self-esteem, future orientation, positive mental health, mattering, and personal identity (Fitzgerald et al., 2017; Hatala et al., 2017; Ulturgasheva et al., 2014). Four studies listed multiple definitions for resilience (Isaacson, 2018; Kral et al., 2014; Goodkind et al. 2012; Sasakamoose et al., 2016). Table 1 provides an overview of resilience definitions in terms of how they related to different ecological levels.

### Micro-System Perspectives of Resilience

In Indigenous-specific approaches, resilience was identified as “beyond health,” and more closely aligned with an individual’s intrinsic spirit and hope (Bruner et al., 2019; Kral et al., 2014; Victor et al., 2016). Looking at intrinsic factors/strategies (Table 2), youth who reported high-self-esteem, optimism, positive cultural identity, sense of belonging, sense of accomplishment, taking personal responsibility, and self-reliance, all had higher resilience when faced with stressors (Ames et al., 2015; Baldwin et al., 2011; Barnett et al., 2020; Clark et al., 2013; Gray et al., 2019; Hatala et al., 2017; Harder et al., 2015; Kenyon & Carter, 2011; Mohatt et al., 2011; Fraser et al., 2015; Stumblingbear-Riddle, 2012; Tiessen et al., 2009; Ulturgasheva et al., 2014; Ungar et al., 2008; Wexler et al., 2014). Definitions from micro-system perspectives on resilience are found in Table 2.

**Table 2:** Definitions important to the understanding of intrinsic resilience factors and strategies

Resilience strategy	Definition
Self-esteem	A belief in yourself; being comfortable the way you are (Ames et al., 2015; Barnett et al., 2020; Gray et al., 2016, 2019).
Optimism	A positive outlook toward the future (Ames et al., 2015; Hatala et al., 2017).
Positive cultural identity	A feeling of personal pride being associated with an Indigenous community and its cultural practices (Clark et al., 2013; Gray et al., 2016; Wexler et al., 2014).
Belonging	A feeling of comfort and support in your environment and community (Barnett et al., 2020; Hatala et al., 2017; Kenyon & Carter, 2011).
Accomplishment	Being able to learn and perform new skills. These can be both culturally important skills like hunting, or societally important skills like getting a new job, and being able to cook (Gray et al., 2019).
Self-reliance	Being able to confidently provide for yourself and find support that is necessary to work through problems (Ulturgasheva et al., 2014; Wexler et al., 2014).

### Meso-System Perspectives of Resilience

Indigenous-specific perspectives valued relationships among family, extended family, clan, and nation. Relationality was considered to other people, as well as to the natural environment. Common Indigenous-specific concepts included connectedness, reciprocity in relationships, spirituality, relationships to the land, balanced relationships (e.g., Medicine Wheel teaching; Ritchie et al., 2015), and collective healing (Isaacson, 2018; Kral et al., 2014; Sasakamoose et al., 2016; Freeman, 2017; Goodkind et al., 2012; Isaacson, 2018; Kral et al., 2014; Mohatt et al., 2011; Ruttan et al., 2008; Rasmus et al., 2014; Wood et al., 2018; Baldwin et al., 2011; Barnett et al., 2020; Gray et al., 2019; Hatala et al., 2019; Hatala et al., 2017; Kral et al., 2014; McMahon et al., 2013; Mohatt et al., 2011; Wexler et al., 2014). Caring for nature was a theme that appeared less frequently, despite Indigenous youth being at the forefront of many Indigenous environmental advocacy efforts (Ritchie, 2021). By spending time in nature, Indigenous youth who lived far away from their home communities reported feeling a greater sense of connection with their homelands (Gray et al., 2019; Hatala et al., 2020; Morton et al., 2020; Kral et al., 2014; Rasmus et al., 2014). Nature also modeled resilience interactions and relationships for the youth (Hatala et al., 2019, 2020; Mohatt et al., 2011; Strickland and Cooper, 2011; Ulturgasheva et al., 2014). By seeing the seasons change, continual growth, and interdependence of natural elements and watching animals interact with each other, Indigenous youth reflected on change, adaptation, and the cycle of renewal (Hatala et al., 2019, 2020; Morton et al., 2020; Ritchie et al., 2015). Additionally, youth reported that being in nature generally calmed them and gave them a sense of peace and re-establishing personal control of emotionality (Hatala et al., 2020; Fraser et al., 2015).

While cross-time trajectories are not evident in research, some studies considered pathways to resilience, from micro- and meso-system perspectives. A study by Fitzgerald et al. (2017) used path analytic statistics to test a causal model, from suicidal ideations or a path to resilience in a sample size of 3,446 Indigenous youth. This model identified that suicide attempts decreased when youth (both male and female) had supportive adult relationships (i.e., adults that believed in their success, listened to youth, cared about where youth were when they were not at school/home) which predicted youth resilience (Fitzgerald et al., 2017). Another model identified pride in cultural identity and positive community associations to be the most predictive of youth resilience (Gray et al., 2016). Specifically, pride in identity led to an increase in self-esteem of 5% (CI: −2% to +21.4%), while positive community associations led to an average increase in self-esteem of 22.6% (CI: +0.9% to +44.3%; Gray et al., 2016).

### Macro-Perspectives of Resilience

Indigenous definitions of resilience placed an emphasis on community-based systems of resilience including the following: healing practices like traditional medicine (e.g., plants, practices such as attending longhouse, sweat lodge, healing dances), traditional teachings (e.g., Creation stories, Thanksgiving address, 7 Grandfather Teachings, planning for the next 7 generations), Native language learning, and the process of forming meaningful relationships (Barnett et al., 2020; Hatala et al., 2019; Mohatt et al., 2011; Sasakamoose et al., 2016; Ungar et al., 2008; Wexler et al., 2014; Wexler et al., 2016).

Despite the breadth of pre-specified factors covered, some studies decided to allow youth participants to create their own definitions of resilience (Fitzgerald et al., 2017; Hatala et al., 2017). This allowed for youth to create personalized resilience models and account for differences in cultural interpretations of resilience (e.g., self-mastery, cultural connectedness).

### Challenges to Resilience

Despite the heterogeneity in Indigenous communities studied, there were significant similarities in stressors. General stressful life events such as changing schools, bullying, poverty, pregnancy, death of loved ones, and peer/family suicide were prominent youth responses when asked about stressors (Baldwin et al., 2011; Hatala et al., 2017; McMahon et al., 2013; Ulturgasheva et al., 2014; Kral et al., 2014; McMahon et al., 2013; Sasakamoose et al., 2016; Snowshoe et al., 2017). Studies that indicated risk factors did not ask youth participants to expand on their rationale for selecting a particular life event and, instead, had participants either spontaneously list or discuss these (Goodkind et al., 2012; Isaacson, 2018; Kral et al., 2014; Krieg, 2016; Rasmus et al., 2014; Strickland and Cooper, 2011; Wexler et al., 2014), or have youth select from a list of pre-specified stressors (Baldwin et al., 2011).

Where sex differences were considered, females reported experiencing significantly more stress than males (Baldwin et al., 2011; Fitzgerald et al., 2017; Gray et al., 2016). Females reported a higher incidence of depression and lower self-esteem, as measured using the New Mexico Youth Risk and Resiliency Survey (Fitzgerald et al., 2017) and Rosenberg’s Self-Esteem (Gray et al., 2016; Wexler et al., 2014) scales, respectively. Some studies linked this to the potentially higher rate of violence and sexual abuse that Indigenous women may face (Gray et al., 2016; Wexler et al., 2014). Drug and alcohol dependence/use was noted in several studies (Baldwin et al., 2011; Hatala et al., 2017; McMahon et al., 2013; Mohatt et al., 2011; Ulturgasheva et al., 2014; Wexler et al., 2014; Harder et al., 2015; Krieg, 2016; Rasmus et al., 2014; Wood et al., 2018). One study emphasized self-medication as a key challenge to resilience, with youth depending on drugs or alcohol in attempts to cope with chronic stressors in their life (Strickland and Cooper, 2011).

Although a strong familial presence generally provided Indigenous youth with the strength to persevere, sudden changes in the family dynamic, parental unemployment, the intergenerational transmission of trauma, and lack of parental support played a role in challenging Indigenous youth resilience (Baldwin et al., 2011; Hatala et al., 2017, 2019; Strickland and Cooper, 2011; Ulturgasheva et al., 2014; Wexler et al., 2014). Lack of communication, attributed to intergenerational trauma, contributed to a disconnect between Indigenous youth and Elders and their communities (Clark et al., 2013; Goodkind et al., 2012; Hatala et al., 2020; Ungar et al., 2008).

Systematic racism was commonly reported by youth (Clark et al., 2013; Hatala et al., 2017; Njeze et al., 2020; Victor et al., 2016; Wexler, 2014; Wood et al., 2018) (Table 3). Racism led to difficulty in youth interacting with others outside of their community, shame connected to their cultural identity, and trouble with law enforcement (Goodkind et al., 2012; Hatala et al., 2017; Strickland and Cooper, 2011; Krieg, 2016). While most youth recognized that their ancestors had to contend with hardship due to colonial impacts, some studies found that youth themselves did not believe that they were still impacted by historical factors (Goodkind et al., 2012; Hatala et al., 2017; Ulturgasheva et al., 2014). One study suggested that Indigenous youth may feel entrapped by the cycles of adversity (i.e., substance abuse, poverty) and trauma narratives (Krieg, 2016).

**Table 3:** Data extracted from studies

Source	Population	*N*	Study design	Definition of resilience used by study	Resilience measures	Challenges to resilience	Resilience factors	Resilience strategies
Ames et al. (2015)	- Aboriginal - Off-reserve	283	Quantitative	N/A	- Change in depressive symptoms, optimism, and self-esteem	N/A	- High self-esteem and optimism, less at risk for depression	-Not specified
Baldwin et al. (2011)	- American Indian - White identity - Highschool students - Off-reserve	221	Quantitative	Social support, cultural identity, and other contextual influences. Multifaceted	- Likert scale was used to measure agreement with statements on social support, family, and peer influence	- Stressful life events such as entering high school/school transfer - Pregnancy, family member attempts suicide, adult of importance has alcohol or drug problem - Verbal abuse, parent unable to find employment gossip about a friend’s attempt suicide, serious argument with a friend - Break up with significant others etc. - Females are more likely to be depressed due to more stressful life events	- Cultural identity was not associated with increased risk of substance use of risky behavior directly - AI identity positively related to proactive family and peer influence. This influence mediates risky behaviors. - Helping to identify culture as a protective factor	- Maintaining cultural heritage practices showed increased health - Programs to promote resilience should involve the whole family and attempt to do so in a culturally appropriate way
Barnett et al. (2020)	- Alaska Native Youth	111	Quantitative	Community and cultural factors leading to the formation of a supportive environment for positive mental health	- Survey rating positive and negative affect schedule, problem-focused cultural coping, interpersonal needs, how much youth matter to others, and self-esteem	- Gender - Colonialism, historical, and current trauma leading to suicidal tendencies	- No significant change seen in the perception of mattering to others, self-esteem, or perceived support for coping with life stressors from friends or family - More positive mood, increased sense of belongingness, greater perceived internal ability to handle potential life stressors after attending camp	- Cultural camp allowed youth to develop connections with peers, a sense of self and belonging
Bruner et al. (2019) Hand searched	- Indigenous youth who took part in a sharing circle	99	Qualitative	Optimal youth development from an Indigenous perspective typically goes beyond healthy emotional and mental development to include other aspects of the person (e.g., body and Spirit)	- Medicine circle—physical, emotional, cognitive, spiritual well-being	N/A	- Balance, developing positive attributes, learning about oneself through sport and physical activity - Connecting with peers, friends, community, coaches, the land, and the Creator - Role of supports in their participation and development	- Positive cultural impact of sport and physical activity engagement on identity development, and learning about health, and wellness
Clark et al. (2013)	- British Columbia Indigenous youth	40	Mixed methods	N/A	- Talking circles and surveys determined youth’s pride in their ancestry, connection to ancestry, the experience of racism, and health care	- Labeling of youth who go to a mental health counselor - Distance from elders - Racism impacts on sense of self	- Positive identity with culture - Ability to speak Indigenous language led to more involvement in ceremonies	- Importance of making youth more widely connected with Indigenous healing approaches (sweat lodge and medicines) - Connection with elders- not as strict as parents, learn more about culture
Fitzgerald et al. (2017)	- AI/AN youth in New Mexico	2794	Quantitative	Factors associated with a reduced risk of suicide	- Survey questions for protective factors on language, parent belief in student, adult involvement in life, suicide attempts	- AI female higher risk of suicide than AI boys - AI in general higher risk than normal population	- Final models indicate that positive relationships with adults at home, school, and in the community remained significantly protective for girls, whereas for boys only relationships with adults in the home remained protective	- Not specified
Fraser et al. (2015) Hand searched	- Inuit youth living in a residential facility in Montreal	13	Qualitative	N/A	- Interview questions about: - Trusting relationships - Space for communication/expression - Structure - Skills - Personal space - Attention - Respect - Responsibility/accountability - Having positive experiences - Sense of control over own life - Speaking one’s language - Cultural activities - Connection with family/positive relationships - Ability to transmit culture -Outdoor activities and space -Feeling normal -Spirituality	- Out-of-home/community placements - Broken intergenerational transmission of parenting skills and culture	- Needs - Culture - Structure - Family, and family relationships spending time with family - Identify family as role models - Cultural activities especially making food talking to Elders reflection on previous generations - Natural environment - Sense of control - Feeling normal	-Not specified
Freeman (2017) Hand searched	- Haudenosaunee youth	14	Qualitative	Indigenous-based resilience is innate, spiritual, and is relational to the land and environment	- The pride of an individual’s identity. - Understanding of the world and a sense of purpose in life.	- Historical trauma, colonialism - Poverty	N/A	- Social agency in the form of activism, guided by cultural practices
Goodkind et al. (2012)	- Dine youth	14	Qualitative	Multiple definitions for resilience—overcoming stress, social support, positive adaptation, community resilience	- Open-ended questions leading to coded themes	- Historical trauma—youth did not have knowledge of their history, but had little belief that historical trauma affected their own lives past that of loss of traditions. - Lack of ability to communicate with elders - Sadness, behavioral issues among youth, and distrust of outsiders	- Families and Elders are viewed as symbols of resilience pushing through hard times in the past and keeping the tradition alive	- Most youth mentioned talking with friends or family as a primary method of coping - Potential interventions should involve intergenerational teaching on beliefs in a culturally appropriate, facilitated way
Gray et al. (2016)	- Inuit youth	452	Quantitative	N/A	- Survey questions identifying pride in identity, participation in culture, social support, emotional demands, education, and income	- 21% reported suicidal ideation in the last 12 months. - Young women have higher suicides, and lower self-esteem than young men as there are higher rates of violence and emotional demands placed on them.	- Greater mental wellness was associated with greater pride in Inuit identity and more frequent harvesting of animals, a strong relationship to the land, sharing, and consuming of traditional food - Some evidence of higher self-esteem among youth with greater collective pride in Inuit identity	- Lower prevalence of suicidal ideation among youth that shared food - Greater mental wellness in communities with more positive interaction, more emotional support
Gray et al. (2019)	- AI youth - North Plains tribes	56	Mixed methods	The ability to “bounce back” and change for the better	- Focus group and youth personal balance tool divided into a tool similar to that of the Medicine Wheel to learn what hope looks like to participants	N/A	- Connecting with nature and AI identity - Mastery of skills and gifts, self-esteem, accomplishments, happiness, and enjoyment, impulse control, sensitivity, forgiveness - Generosity; problem-solving; wisdom; freedom from fear, hate, jealousy, and other negative emotions, and behaviors; commitment to lifelong learning and service; and doing things in moderation	- Trusting and supportive relationships among youth. - Family-oriented approach leads to the greater building of healthy relationships - Validation of individual personal growth through goal setting - Opportunities for youth to take leadership roles
Harder et al. (2015) Hand searched	-Indigenous youth living in BC Carrier Sekani community	130	Mixed methods	N/A	-Changes in depression, hopelessness, suicidal ideation, and self-esteem - Cultural awareness, connection, identity - The Beck Depression Inventory-II - The Beck Scale for Suicide Ideation	- Drug/alcohol abuse - Physical, sexual, mental, emotional abuse - Cultural disconnect - Lack of healthy activities - Depression, hopelessness, and suicidal ideation	- Culturally appropriate and culturally specific activities - Higher self-esteem - Sense of belonging and identity - Beck scales decreased when children participated in culturally appropriate activities by approximately 3 points	- Not specified
Hatala et al. (2019)	- AI youth Plains Cree and Métis in Canada - Urban	38	Qualitative	Notions of reciprocity, spirituality, Indigenous knowledge	- Coded Interviews	- Lack of reciprocity and caring in home life	- By interacting with the land and nature, youth comment on the ability to find connections between the “bush” and what they call home. Interactions with land calming.	- Gift giving as form of individual acts of life, and community - Focus on spiritual principles - Intimacy and interrelatedness with land and Nature, as kinship relations and family type bonds
Hatala et al. (2020)	- Indigenous youth - Plains Cree and Métis	28	Qualitative	Positive adaptation and resistance in the face of colonization, historical traumas, or structural violence, as well as current stresses, challenges, and demands	- Digital cameras and open talking circles - Talk about meaning and interpretations of photos to create categories - (1) Nature as a calming place; (2) building metaphors of resilience; and (3) providing a sense of hope	- Lack of close family - Physical burden of cold on body and emotions	- Meaning-making in the land. Interactions with Nature lead to traditional creativity and storytelling instilling inner peace and decreasing anger. - Youth recognized that similarly to seasons there will always be high and low points in life, but things are constantly changing and growing - Nature was seen to model relationships, situations in life, and provide a sense of reassurance and hope for youth	- Being in nature - The feelings of calming serenity which nature offers cannot be derived by attending support programs alone, socializing with friends, or family, or even participating in cultural activities and school initiatives
Hatala et al. (2017)	- Indigenous youth - Plains Cree and Métis	28	Qualitative	Belonging, self-mastery, or cultural identity and continuity	- Two-eyed seeing framework using open-ended questions made in collaboration with Indigenous partners to determine the cultural connection, connection to community, views of the future	- Colonization and historical trauma - Inner-city environment—increased poverty, daily, and persistent microaggressions, marginalization, family violence, sexual abuse, social, and cultural dislocation, peer violence, threats of gangs prostitution, substance abuse, self-harm, and suicide - Childhood “taken” - Sense of identity lost as children because family roles were inconsistent	- Confidence and safety in the future leads to better self-mastery skills - Plan for the future giving a sense of positionality in time - More cultural activities in a community lead to less suicide. - Positive cultural identity—more self-worth, self-efficacy connectedness, and purpose	- At least one social support with consistent responsiveness - Grandparents were crucial resilience promoting resources
Isaacson (2018)	- Native American youth from Plains tribe	8	Qualitative	Multiple definitions for resilience listed—keywords include: internal strength, community connections, spirituality, harmony, facing challenges, reducing negative stress, strong identity	- Herth Hope Index - Child and Youth Resilience Measure, Child Version [CYRM-28]	- The concept “walking in two worlds” which is described as trying to maintain one’s physical, mental, emotional, and spiritual well-being while maintaining a level of assimilation with the mainstream culture - Desire to learn the traditional language and cultural roots, but limited opportunities to do so.	- Knowing and participating in traditional cultural activities, including language proficiency	- Traditional cultural practices such as working with the horse, enhance ethnic identity due to the horse’s historical and present-day connections to many NA/AI peoples
Kenyon & Carter (2011)	- AI youth - High school - Northern Plains	95	Quantitative	N/A	- Survey questions monitoring demographics, ethnic identity, sense of community, positive affect, feelings of depression, psychosomatic symptoms	N/A	- Ethnic identity and sense of community promote membership and create a feeling of emotional safety with a sense of belonging to a large collective	- Being actively involved in cultural practices helped to foster a sense of community in those with lower internal ethnic identity
Kral et al. (2014)	- Inuit youth from elementary and high schools in the community of Igloolik, Nunavut, Canada	23	Qualitative	Multiple definitions for resilience are listed—keywords include: hope, being grounded, spirituality, one’s self-being part of nature, the land, healing	- A structured interview used across sites by youth to describe what matters to them and what is at stake for them in terms of challenges and successes	- The most common source of stress reported by Inuit youth was school - Bullying and not attending classes were the most prominent problems identified	- Individual resilience is closely connected to family and community resilience, reflecting the role of relational, ecocentric, and cosmocentric concepts of self	- Talking to a friend about problems involving relationships with other youth, romantic issues, and family conflict - Talking to a parent or adult caregiver as a coping mechanism for specific problems - Being on the land, usually with family, was also a source of strength - Youth engagement in the community through programs, and activities
Krieg (2016)	- Urban Indigenous First Nation and Métis	6	Qualitative	N/A	- Participatory Action Research (PAR) method called Photovoice (a grassroots community assessment tool that enables local people to identify and represent their community through the use of photography as the medium)	- The effects of residential schools - Participants felt that the cycle of adversity (addictions, abuse, and poverty, and in areas like academic achievement and employment) was a difficult challenge - Stereotypes, and racism towards Indigenous women - Struggles in learning about their culture due to feelings of shame of negative stereotypes - Lack of cultural programming	- Cultural continuity creates positive and lasting change for Indigenous people, families, and communities - Motivation and support from family and peers - Positive female Indigenous role models	- Connecting and reconnecting to traditional teachings was essential for Indigenous youth - Cultural programming specific to Indigenous girls was key to their social, emotional, and sexual development
McMahon et al. (2013)	- AI youth - Northern Plains	95	Qualitative	The strengths of AI youth, personal attributes, positive relationships, and AI culture	- Open-ended survey with coded theme responses	- Many individuals commented they did not face any challenges (32.6%) Dropping out of high school, teen pregnancy, and substance abuse as common sources of struggle in their personal lives - Some youth stated that they did not believe that there were any community strengths	- The frequency in which youth cited loving “everything” about their lives (10.5% of total responses) is also noteworthy. - Overall, the positive aspects referenced by the youth are consistent with traditional AI values of collectivism and community	- Family support - Attending pow wows
Mohatt et al. (2011)	- Alaska Native - Rural community	284	Quantitative	A holistic sense of connectedness of the individual with their family, community, and environment.	- Awareness of Connectedness Scale - Alaska Native Cultural Identification - Reasons for Life - Multicultural Mastery Scale	- Substance use	- Greater connection with cultural leads to an internalization of cultural specific values such as reciprocity and caring for each other	-Not specified
Morton et al. (2020)	- Plains Cree and Métis youth	28	Qualitative	N/A	Coded analysis of images and youth explained the physical representation of resilience	- Loss of connection to nature as youth age - Less connection to culture and ceremonial activities	- Being in and with nature—functions as a metaphor for youth resilience—healing in the fall - Nature functions as a source of health and well-being embody for mental health Spiritual source - Water teachings—woman and the relationship to the Creator	- Cultural elements that evoke spirituality and survival Practicing gratitude - Being in nature, learning from animals
Njeze et al. (2020)	- Indigenous youth - Canada	28	Qualitative	Reduced vulnerability to environmental risk experiences, the overcoming of stress, or adversity, or a relatively good outcome despite acute distress	- Stories, photos, and field notes, interview transcripts were analyzed - Sharing circles, photovoice, conversational, and photo-elicitation interviews, and naturalistic interactions	- Acute hardship (e.g., abandonment, victimization, racism), difficulties, and/or periods of sustained environmental stress (e.g., poverty, discrimination)	- Strong cultural identity and family connections - Positive Outlook	-Engagement in social groups - Doing community service - Practice of the arts
Ruttan et al. (2008) Hand searched	- Homeless young women in Edmonton	18	Mixed methods	In order to survive the situations related to their homelessness, youth activate many strengths; they use connections formed on the street and those of the community	- Historical, ecological, social, cultural factors related to their homelessness	- Historical trauma/colonization	- The impact of residential schools—intergenerational trauma contributes to homelessness - Relations with child welfare - Knowledge about their background; connection to their culture	- Health-promoting narratives reinforced in community-based healing programs that acknowledge and address racism and systemic barriers.
Pertucka et al. (2016) Hand searched	- Buffalo First Nations youth	78	Mixed methods	N/A	- Youth and Elder teams to explore cultural practices that may inform the youth’s paths to living well. - This process included engagement, module creation, co-delivery, and knowledge sharing	N/A	- Positive attitudes, and activities	- Elder-youth relationships - Leadership development - Emphasis on learning with the land - Language learning
Ranahan and Yuen (2017) Hand searched	- First Nations Youth	14	Qualitative	N/A	- Healing - Life - Ceremony - Relationships - Hope	N/A	- Learning from elders is critical to young people’s hope - Belonging, acceptance, and physically being with other people - Youth view life as a journey made up of ever-changing moments in time - Living in the present, looking towards the future, while holding the past	- Engaging in a ceremony in the present, the youth were able to look forward to the future by holding onto these past traditions - Embodying resilience: laughter, physical activity, eliciting laughter, and a connection to the land
Rasmus et al. (2014)	- Yup’ik Alaska Native youth	25	Qualitative	A dynamic process involving people, events, and settings sharing relationships, linkages, interactions, and transactions that distribute and transform resources	- Interviews through a three-tiered qualitative method	- Dangers at home, school, or somewhere else within the confines of the village community - Staying away from alcohol and marijuana - Personal setbacks and trauma - Collective experience such as disease, natural disaster, and historical, and ongoing colonization	- Formal and informal community structures that support hunting and other traditional subsistence activities	- Community and kinship relationships - Playing sports (e.g., basketball) - Taking care of other people - Traditional diet - Remembering or thinking about people you love or people that love you
Ritchie et al. (2015)	- Youth from Wikwemikong Reserve	43	Mixed methods	The ability to successfully cope with change and misfortune	- 14-item Resilience Scale - Journals, interviews, talking circles, and Elder Teachings	N/A	N/A	- Connecting with ancestors, culture, community - Connecting to Creator, and other elements in Nature - Practicing Medicine Wheel teaching
Sam et al. (2015)	- Indigenous students in British Columbia	136	Quantitative and qualitative	A person’s ability to overcome adverse life events	- 14-Item Resilience Scale+ Demographics, Socioeconomic status - Attachment styles assessed using the Relationship Questionnaire	- Life-course that is filled with stressors or obstacles that disrupt healthy development and contribute to poorer resilience-oriented outcomes, insecure attachment styles, and diminished mental health, and well-being	- Attachment security related to resilience - Preoccupied attachment style with lowest resilience - Positive parental characteristics (e.g., higher education) with consistent caregiving and appropriate discipline practices	- Not specified
Sasakamoose et al. (2016)	- First Nations and Métis youth	14	Qualitative	Multiple definitions for resilience listed—keywords include: navigate resources, positive adaptation, cultural values, family, cultural connectedness	-Six research questions guided the research design	- Trauma due to colonization and residential schools - Health disparities - Suicide - Poverty	- Holistic health - Cultural knowledge (spirituality, traditions, identity) - Sports access - Relationships with elders and role models	- Cultural activities (beading, dancing, drumming, singing, etc.) - Navigating addictions
Snowshoe et al. (2017)	- First Nations, Métis, and Inuit youth	290	Quantitative	N/A	- Cultural Connectedness Scale-Short Version - Demographics - Stressful Life Events - The BC Adolescent Mental Health Survey-Fourth Edition - The Hemingway Measure of Adolescent Connectedness-Short Version - The MAC 5-A-Short Version - The Satisfaction with Life Scale for Children	- Stressful events (e.g., loss of a close friend or family member, police interaction, and social services interaction) - Life stressors	- Positive family and school connections - Cultural knowledge (identity, traditions, spirituality)	- Developing cultural connectedness
Strickland and Cooper (2011)	- Pacific Northwest on reserve - High school students	30	Qualitative	N/A	- Coded measure of appearing themes from interviews	- Getting into trouble - Singled out for being Indian - Treated unjustly by police - Expelled for getting into fights - Living far away from school and fear of being late - Changing family situation - Preventing gossip and navigating relationships with Indian identified and non-Indian identified peers led to competing values	- Selecting friends appropriately that did not promote, “getting in trouble” behavior, or gossip - Blowing off steam and going to parties was seen as helpful, but had the potential to lead to more harm than good - Community and family as a source of support	- Attending Pow Wows - Receiving education tutoring provide - Land-based activities - Praying, and talking to elders - Sharing of resources
Stumblingbear-Riddle (2012)	- Urban AI adolescents - AI from the Kiowa tribe	213	Mixed methods	A “dynamic process that enables the individual to respond or adapt under adverse situations”	- Native American Community Health Survey: Youth (modified) - American Indian Enculturation Scale (modified) - Tri-Ethnic Center’s Self-Esteem Scale (modified) - Satisfaction With Life Scale (modified) - Perceived Social Support from Family and Perceived Social Support from Friends	- Unresolved emotional distress due to the impact of colonization - Lack of cultural connection - Increased suicide risk - Increased levels of hopelessness - Limited tribal support systems and resources - AI adolescents are often torn between two cultures (i.e., AI and Euro-American) No opportunity to regularly engage in AI cultural activities	- Higher levels of enculturation demonstrated higher resilience - Self-esteem - Supportive friendships	- Tribal cultural activities
Tiessen et al. (2009) Hand searched	- Indigenous youth in Northern Manitoba	N/A	Mixed methods	N/A	- Collective efficacy, social capital, and communal control/mastery	- Governmental policies of assimilation and colonization over many centuries (lost control)	- Results suggest an association between greater perceived individual internal (“Self”) control and greater psychological well-being - Results suggest a relationship between perceived group internal control and greater psychological well being	-Not specified
Trout et al. (2018)	- Inupiaq Alaskan Native youth	N/A	Qualitative	N/A	- Photovoice - Digital storytelling projects - Interviews	- Loss of traditional ways, which is endangering the continuity of Inupiaq culture and identity - Historical trauma - Culture and identity loss - Two cultural worlds—White/Western versus traditional/Inupiaq - Future aspirations	- Inupiaq language immersion education	- Adaptability, development, and change
Ulturgasheva et al. (2014)	- Alaska, Canada, Norway, and Russia—*only data from Alaska and Canada used* - Inuit and Alaska Native	N/A	Qualitative	Resilience is not a linear process	- Interview with identified themes	- School bullying—intergenerational conflict - Domestic violence - Substance abuse - Being neglected - Parent’s drinking - Breakups - Boredom - Suicide - Not getting to practice culture	- Self-reliance - Sense of responsibility and competence in activities - Supportive friendships	- Talking to friends and family - Practicing culture on the land, hunting, and fishing - Talking to older community members - Sharing resources
Ungar et al. (2008)	- Urban Aboriginal youth in Halifax and Winnipeg, Sheshatshiu, Innu	19	Qualitative	An individual’s capacity to cope with adversity. Also, the capacity of the person’s community to provide the health resources necessary to nurture and sustain well-being, providing individuals opportunities to access health resources in culturally relevant ways	- Coded theme structure interview	- Access to material resources - Access to supportive relationships - Development of a desirable personal identity - Experiences of power and control - Adherence to cultural traditions - Experiences of social justice - Experiences of a sense of cohesion with others	- Supportive relationships - Power and control over their own life - Flexible adherence to cultural traditions	-Not specified
Victor et al. (2016) Hand searched	- First Nation youth, southern Saskatchewan	14	Qualitative	Kiskenimisowin is the Cree word for “knowing oneself.” One comes to know oneself through interaction with the self (introspection, self-reflection) and the world around oneself	- Qualitative examination of art and conversations recorded in field notes	- Oppression, ongoing colonization, and racism	- Respectful relationships - Cultural values - Cultural and individual identity - Cultural safety	- Creative forms of communication - Using art to create a safe space
Wexler (2014)	- Alaska Native adults and youth; 14–21	9	Qualitative interviews	Resilience involves acute hardship (e.g., victimization) and/or forms of sustained stress (e.g., poverty, discrimination), and—despite these risks—results in positive or unchanged behavioral and/or health outcomes	- Qualitative coding	- Historical trauma and racism - Suicide, peer deaths, suicide attempts, peer/parental drinking, or fighting, removal from homes - Community problems were being seen as individual issues - Awareness of historical discrimination	- Sense of connectedness - Sense of belonging in home communities - Cultural identity and affiliation	- Community activities that promote connectedness - Engagement in culture through traditional storytelling
Wexler et al. (2016)	- Alaska native boys and girls between 10–20	341	Qualitative	A result of the strengths and resources available to youth, within their family, community, and culture. Risk must be understood in the context of young people’s lives. If there are enough protective resources in place, young people may thrive despite the risks	- Demographic questionnaire, 40 items about life, family, community, friends, values, and participation and attitudes to culture Likert scale of agreement - 3 open-ended questions about the community and youth’s life -3 questions about how youth deal with problems	N/A	- Belief in self and reliance; ability to work through problems - Sense of responsibility; ability to take responsibility for action and do the right thing - Supportive family - Supportive peers - Supportive community - Positive responses about decision making, perceived strength, and future outlook	- Engagement in cultural activities
Wexler et al. (2014)	- Alaska Native youth - Northwest Alaskan Inupiat community	20	Qualitative interviews	The strategies used to overcome acute and ongoing difficulties, with an emphasis on understanding the emic meanings within the contexts of resilience and risk, and the dynamic relationship between individuals and their environments	- Coded theme from semi-structured interviews to identify everyday experiences	- Losing relationships with close family, and friendships - Lack of comfort accessing parents for support when having conflict with friends or peers - Girls experienced higher hostility and bullying from outsiders - Boys were more likely to get into physical altercations and received advice to fight back - Fewer fun activities - Boredom	- Parental support - Youth were relatively self-reliant and creative in problem-solving - Sense of competence - Protecting and helping others contributed to a sense of well-being, particularly in boys	- Reciprocity within a peer group for problem-solving, peers would offer help, and advice knowing that cultural norms dictate that they will be helped when they are in need - Family teaching and learning was protective for young girls allowing - Giving back to the community
Wexler et al. (2013)	- Alaska Inupiat youth	20	Qualitative interviews	Resilience—resources/activities drawn upon to manage, overcome, and/or effectively handle challenges. Youth resilience refers to the developmentally specific capacity to navigate ongoing difficulties.	- Coded interviews	- Losing connectedness/contact with cherished others - Peer pressure for substance use	- Biological relationships - Connectedness, sense of belonging - Peer relationships	- Nurturing relationships with people whom they believed were good to know - Engagement in culture through community ties and kinship
Wood et al. (2018)	- Native youth on and off the reservation	22	Qualitative interviews	Survivance is purposely a broad term used to capture the unique kind of cultural change and survival in Indigenous life. It is used as an alternative to narratives of merely “holding on” or “being resilient” to instead describe the way Indigenous communities are generative in how they adapt Indigenous culture and modes of being to contemporary circumstances and dominant culture	- Coded interviews	- Familiar and community struggles with mental and physical health - Exposure to substance abuse - Issues in accessibility to healthcare clinics, healthy food, employment opportunities, isolation, and boredom - Lack of preparation for navigating non-Native and off-reservation space - High incidence of historical trauma - Poor mental health; community gossip and lack of privacy, lack of motivation to be successful in life -Stereotypes and stigmas	- Reservation facilitates feelings of comfort, belonging, and social support, feelings of freedom, and safety - Cultural traditional knowledge - Youth empowerment - Having intergenerational relationships	- Participation in language/cultural classes - Connecting to family - Giving back to the community
Yeh et al. (2015)	- Samoan middle and high school students from San Francisco	58	Mixed methods	The ability to positively adapt to adversity and life stressors	- Multidimensional scale of perceived social support, open-ended questionnaire evaluating the experience of a cultural program	N/A	- Development of skills such as self-expression, persistence, overcoming fears, and teamwork and collaboration. - After school program increased scores of social support, leadership competence, community engagement, and increased resilience - Increased feeling of support, and empowerment in culture due to the building of social connections. - Increased confidence in leadership capabilities. - Love, pride, respect for elders, and family, creativity, and strength were all emphasized factors that youth found helpful	- Indigenous knowledge increased when participating in group - Participants reported that they learned many important strengths of Samoan culture

Culture remains a significant contributor towards resilient living of Indigenous youth. For example, the concept of “walking in two worlds” was reinforced. It was noted that Indigenous youth attempts to maintain physical, mental, emotional, and spiritual well-being, while assimilating to the mainstream culture, this may endanger identity and resilience processes (Isaacson, 2018; Trout et al., 2018). Cultural detachment was identified as a resilience-disrupting process (Isaacson, 2018; Morton et al., 2020; Stumblingbear-Riddle, 2012; Tiessen et al., 2009; Trout et al., 2018; Ulturgasheva et al., 2014; Ungar et al., 2008; Wexler et al., 2013; Harder et al., 2015), leading to loss of connection to, and engagement with, traditional language and ceremonies (Morton et al., 2020; Isaacson, 2018; Trout et al., 2018). Most studies used a strength-based (versus deficit-based) approach, aiming to identify points of resilience that could be proactive and preventative (McMahon et al., 2013). A summary of factors promoting resilience and associated studies are listed in Table 4.

**Table 4:** Factors promoting resilience

Factors	Studies identifying factor
Engaging in cultural activities	Baldwin et al. (2011) Clark et al. (2013) Hatala et al. (2017) Kenyon and Carter (2011) Strickland and Cooper (2011) Ungar et al. (2008) Yeh et al. (2015) Isaacson (2018) Wood et al. (2018) Wexler et al. (2016) Rasmus et al. (2014) Petrucka et al. (2016) Harder et al. (2015) Fraser et al. (2015)
Connection to the land	Gray et al. (2016) Fraser et al. (2015) Ranahan and Yuen (2017) Rasmus et al. (2014) Hatala et al. (2019) Hatala et al. (2020) Mohatt et al. (2011)
Positive personal identity	Ames et al. (2015) Barnett et al. (2020) Gray et al. (2016) Gray et al. (2019) Hatala et al. (2017) Mohatt et al. (2011) Ulturgasheva et al. (2014) Ungar et al. (2008) Wexler et al. (2014) Wexler et al. (2016) Stumblingbear-Riddle (2012) Harder et al. (2015) Clark et al. (2013)
Positive cultural identity	Baldwin et al. (2011) Clark et al. (2013) Gray et al. (2016) Kenyon and Carter (2011) Njeze et al. (2020) Wood et al. (2018) Wexler et al. (2016) Wexler (2014) Victor et al. (2016) Trout et al. (2018) Snowshoe et al. (2017) Sasakamoose et al. (2016) Hatala et al. (2017)
Participation in community program	Yeh et al. (2015) Njeze et al. (2020) Hatala et al (2017) Barnett et al. (2020) Wood et al. (2018) Wexler et al. (2016) Strickland and Cooper (2011) Rasmus et al. (2014) McMahon et al. (2013) Krieg (2016) Freeman (2018) Bruner et al. (2019)
Community relationships	Baldwin et al. (2011) Barnett et al. (2020) Clark et al. (2013) Fitzgerald et al. (2017) Goodkind et al. (2012) Gray et al. (2016) Gray et al. (2019) Hatala et al. (2019) Hatala et al. (2017) McMahon et al. (2013) Mohatt et al. (2011) Njeze et al. (2020) Strickland and Cooper (2011) Ulturgasheva et al. (2014) Ungar et al. (2008) Wexler et al. (2014) Wood et al. (2018) Wexler et al. (2016) Rasmus et al. (2014) Krieg (2016) Freeman (2017) Bruner et al. (2019)

**Fig. 2:**
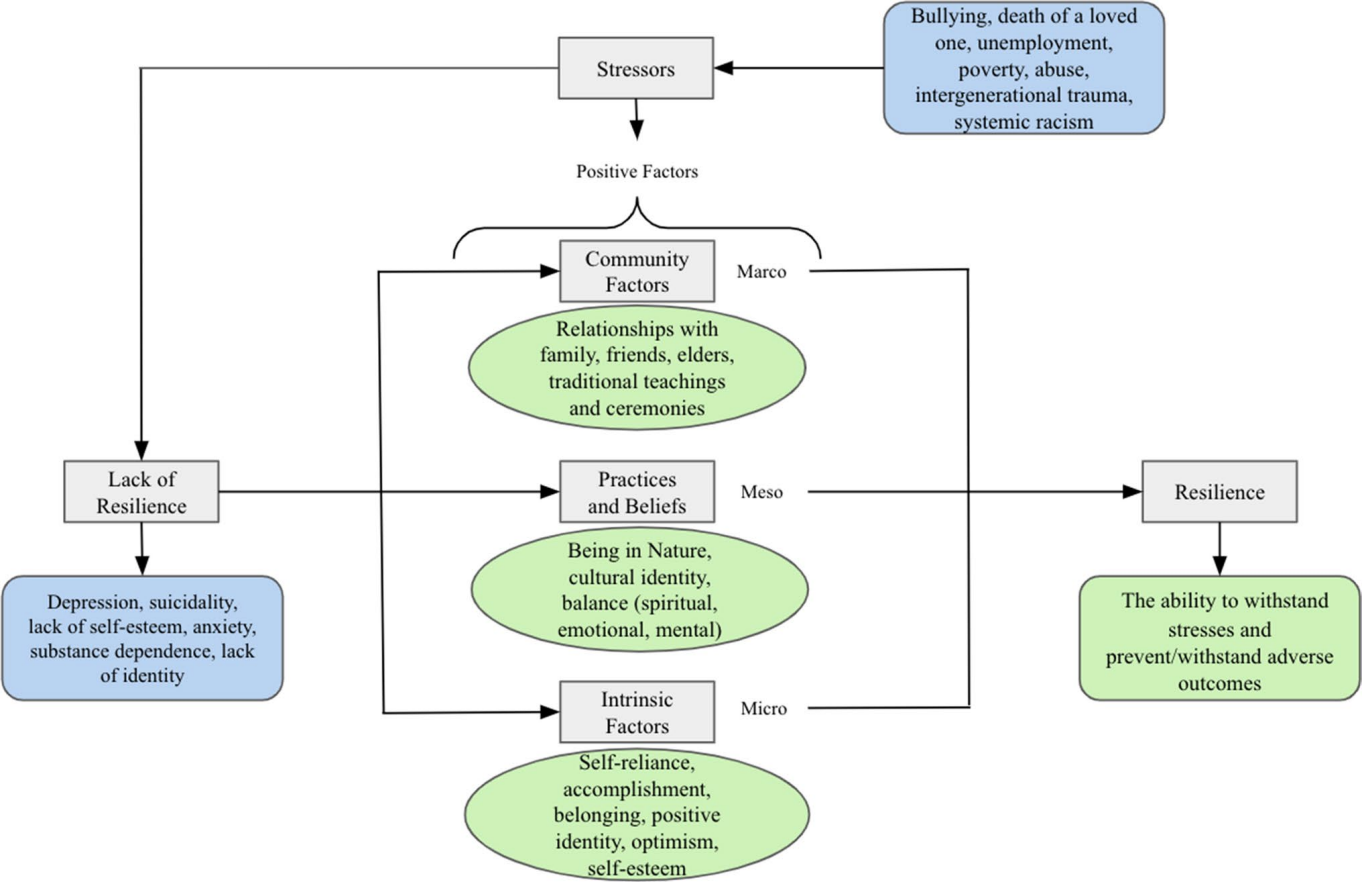
Review of results highlighting resilience process

To summarize these review results on psychological resilience, Figure 2 below points to a core set of interconnected protective factors at the macro-, meso-, and micro-psychosocial environment.

An emerging theme is the importance of physical health, including its correlation with positive well-being in Indigenous youth (Gray et al., 2019; Ranahan and Yuen, 2017). Well-being was heavily impacted by holistic health, access to healthcare, healthy foods, safe drinking water, and engagement in culture-rich community physical activities, such as pow wows (Ulturgasheva et al., 2014; Wood et al., 2018; Strickland and Cooper, 2011; Sasakamoose et al., 2016; Bruner et al., 2019). Similarly, a study examined how a subsistence diet (“living off the land”) promotes physical health, a healthy diet, and a connection to culture (food sovereignty), and contributes to psychological wellness and resilience through the physical, spiritual, and mental dimensions associated with connections to land (Burnette et al., 2018).

## Discussion

Resilience is a complex, multi-faceted process that involves an interplay between stressors, resilience-promoting factors, and pathways. For Indigenous youth, resilience plays a significant role in their lives as they face unique, multi-leveled, and persistent stressors. While most studies note the role of trauma, no studies measured trauma symptoms or experiences directly, or comprehensively included such facets as intergenerational trauma, loss and grief, or ecological anxiety or grief. Some studies considered sex difference, but no studies included in this review considered gender categories, or specifically noted two-spirit youth. This suggests that the definition, meaning, and process of resilience may change depending on the mental health variables studied, as well as the inclusivity of youth groups.

As evidenced by the literature, there are multiple points wherein resilience can be promoted. Cultural continuity, passed between generations, fosters a sense of community connectedness, allowing youth to build and have continuity in their cultural identity. By continuing to practice cultural ceremonies and language transmission, there is continued intergenerational involvement providing opportunities for knowledge sharing, and for youth and elders to become better connected (Clark et al., 2013; Fitzgerald et al., 2017: Strickland and Cooper, 2011; Gray et al., 2019; Yeh et al., 2015). Connection to the land was found to be a significant strategy used in coping with daily stressors and promoting resilience in Indigenous youth (Gray et al., 2016; Fraser et al., 2015; Ranahan and Yuen, 2017; Rasmus et al., 2014; Hatala et al., 2019, 2020; Mohatt et al., 2011). Studies commented on the importance of interactions with nature (Hatala et al., 2019, 2020). Nation-based resilience models were not depicted in these studies, although there are efforts to convey the unique models. For example, Noronha et al. (2021) provide a graphic for a Haudenosaunee wellness model that emanates from the central elements of the Thanksgiving address to specify indicators of resilience factors. Personal growth is indicated by contributions to the community, the study of language, and participation in ceremonies. A “good mind” was indicated by adherence to the Great Law of Peace (peaceability), balanced living (spiritual, physical, emotional, mental), traditional health practices (e.g., medicinal plants), and recreation (e.g., sports, such as lacrosse and dance, such as the jingle dance, considered to function as medicine). This model includes ancestral knowledge and “revealed” knowledge or knowledge providers and figures (e.g., the Great Peacemaker, Tekanawí:ta). Indigenous knowledge is based on oral tradition and person-to-person teaching (e.g., longhouse), where community resilience is prioritized as the route to individual resilience. As Indigenous communities traditionally have a more holistic and community-based sense of health, resilience can take many forms, such as interactions with nature, community participation, clan-based affiliations, and interacting with family and friends. The lessons learned from Indigenous communities are that there is an intrinsic connection and renewal present in nature, community, and culture that promote resilience and well-being. Respectful relationships include self-relating, relating to others, and relating to the environment from a perspective of responsibility and relationship to the Creator.

All reviewed studies found that promoting resilience led to enhanced well-being among youth. Recommendations included integrating community-based or group-based interventions for youth resilience. Despite the heterogeneity in the literature for measuring resilience, allowing youth to express themselves creatively, and allowing the opportunity to explain their process, helped elicit youth-specific resilience factors and strategies. In so doing, participants were not limited to a predetermined or Western definition and could highlight factors that researchers and the literature may not have previously considered. This type of qualitative research allowed flexibility in the discussions surrounding resilience in a field where validated tools for Indigenous youth are minimal (Clark et al., 2013; Ulturgasheva et al., 2014).

As the body of resilience research grows, there will be a need for context-specific measurement approaches, and a close examination of factors that may be different between communities. For example, nation-specific resilience may be derived, in part, from the language that describes aspects of wellness and value-based living. Language learning and ceremonial practices were particularly useful in promoting mental wellness and belonging (Baldwin et al., 2011; Clark et al., 2013; Fitzgerald et al., 2017). Jongen and colleagues conducted a measurement scoping review from Australian, New Zealand, Canadian, and US research (Jongen et al., 2019). They found 20 instruments utilized, mainly from Western resilience measure development. Only three Indigenous instruments were found: Growth and Empowerment Instrument (Australia; Blignault et al. 2016), Cherokee Self-Reliance Scale (USA; Lowe et al. 2012), and the American Indian Enculturation Scale (USA; Stumblingbear-Riddle, 2012). These tapped primarily individual assets and environmental resources, or only environmental resources. Only two measured individual assets, environmental resources and culture-based resilience. In this review, individual assets were considered to be skills (stress management, communication), personal strengths and traits (empathy, personal awareness, optimism), and resilience factors (future orientation, balanced perspective, cultural identity). Environmental resources included support and opportunities, such as peer support, kinship networks, and adult role models. Jongen et al. (2019) suggest that measuring cultural factors, such as cultural connectedness, Indigenous worldviews, and spirituality, may be of greater importance than individual-level factors, as it has not only been linked to mental health and wellness but also improved socioeconomic indicators and academic achievement.

Notably, many studies included suicidal thoughts and depression, a major factor contributing to suicide (Orsolini et al., 2020), in their measurement and definition of resilience outcomes as reduction in symptomatology (Ames et al., 2015; Baldwin et al., 2011; Fitzgerald et al., 2017; Harder et al., 2015; Kenyon & Carter, 2011). Lalonde and Chandler (2009) note that individual and cultural continuity are strongly linked; communities that succeed in taking steps to preserve their heritage, and achieve sovereignty in important areas, are more successful in insulating their youth against the risks of suicide. Cultural initiatives include solution-focused approaches, such as early support to higher suicide-risk sexual minority youth (e.g., Hottes et al., 2016, Pollock 2018), and gender teachings on roles and responsibilities, where all are valued with sacred duties (Anishnawbe Health Toronto, 2017). Mushquash and colleagues advance the public health perspective as central to supporting Indigenous youth resilience, in terms of addressing the social determinants of health, healthy behaviors, and healthy communities (Mushquash et al., 2021). In this view, resilience is a systemic target with resilience-promoting policy (e.g., child and youth mental health, HIV prevention, nutrition, pediatric diabetes prevention, and injury prevention). Local knowledge, community networks, communication networks, and preparedness emerge as important to measure, in addition to individual and community-level outcomes.

As research begins to be put into practice in communities, the results of this review indicate that future research and programming should involve a collaboration of youth and Elders (Bruner et al., 2019; Goodkind et al., 2012). Collaboration between Western and Indigenous resilience research may promote understanding and knowledge sharing for increased well-being for both the youth and community (Fraser et al., 2015). The two-eyed seeing approach is often used as an investigative framework, as opposed to solely Indigenous-science perspectives (First Nations Mental Wellness Continuum Framework, n.d.; Wright et al., 2019). This embraces the collaboration of Indigenous knowledge or “ways of knowing” and Western-based knowledge (Wright et al., 2019). This approach recognizes that Indigenous youth, living on reserve, rurally, or in urban centers, are exposed to Western socialization and engaged in cross-community communications with the greater availability of internet connectivity and presence on social media platforms. Humor, agency in content development, teaching, and representation are resilience approaches used (Loyer, 2020).

There are commonalities among intrinsic and familial resilience factors. Intrinsic factors such as self-esteem, self-expression, and self-efficacy have been observed in many populations of varying economic and racial backgrounds (Gartland et al., 2019; Donnon & Hammond, 2007). Supportive familial and peer relationships associated with a sense of belonging and acceptance were seen as sources of resilience across diverse populations (Fritz et al., 2019; Gartland et al., 2019). Despite this, school engagement and student-teacher relationships were more prominent in Western science than in Indigenous science methods (Gartland et al., 2019; Ungar & Liebenberg, 2013). Future research should compare resilience factors and strategies employed by youth across a diverse array of backgrounds, examining whether they may be inherent despite culture, socioeconomic status, and race.

### Implications for Practice with Indigenous Youth

In examining the complexity involved in a holistic understanding of resilience in Indigenous youth and their communities, it is imperative to consider how these learned strategies can be applied (Blackstock, & Trocmé, 2005; Interrupted Childhoods n.d.). The historical involvement of the foster care system in Indigenous communities emphasizes how the potential to reunite displaced youth with their culture could foster a greater sense of belonging and assist youth in recovering from adverse experiences and stressors connected to their removal from their families and communities (Blackstock, & Trocmé, 2005; Filbert & Flynn, 2010; View of Violence n.d.). Additionally, as organizations strive to promote truth and reconciliation moving forward, this review’s findings highlight the importance of keeping youth immersed in their culture to promote positive future orientations consistent with Indigenous traditions (Blackstock & Trocmé, 2005; Ungar, 2006).

### Study Limitations

One major limitation of our study is the lack of included studies on Native Hawaiian populations. Although “Native Hawaiian” was a search term in our search strategy, only one included review made mention of the Native Hawaiian population and included similar resilience strategies to other Indigenous populations (Yeh et al., 2015). It is of particular note that our search strategy may not have appropriately captured the scope of Native Hawaiian populations due to the inconsistent use of keywords and terminology in publication and countries. Furthermore, the exclusion of Native Hawaiian populations may be due to the overlap of many reviews that include Native Hawaiians and Pacific Islanders in New Zealand and Australia. Due to the differences between traditional and current population boundaries, many reviews including Native Hawaiians did not meet the search strategy or inclusion criteria. To rectify these limitations in future publications, the research team suggests that it may be appropriate to identify specific journals that publish research on specific Nations and populations and to undertake hand searches for a more exhaustive capturing of relevant articles to ensure important groups of study are not left out. Further to this, working with Hawaiian scholars directly would support accessing gray literature, as well as better awareness of existing literature.

Further to this, our inclusion and exclusion criteria limited our included studies to peer-reviewed, published literature. This may introduce bias into the results as published literature is more likely to contain reports of efficacious interventions and studies with positive outcomes. Therefore, by excluding gray literature, conference proceedings, and unpublished literature, there is a potential for publication bias in our review.

Lastly, the broad scope of our search terms may have served as a limitation in our study. By including terms in our search strategy such as “coping” and “cultural intervention” as resilience alternatives, we may have included measures that were too far from our initial overarching definition of resilience. When referring to the results and conclusions drawn from the literature review in this study, it is important to keep these limitations in mind.

### Future Directions

This review explored the extent of the literature on factors promoting resilience in Indigenous youth in Canada and the USA; as noted, Indigenous-specific measurement of youth resilience is a nascent area and important future direction. Due to the general emphasis on truth and reconciliation, it could be interesting to explore cultural resilience factors that can help Indigenous youth who have had cultural fracturation due to foster care involvement (Blackstock & Trocmé, 2005; Ungar, 2006). This specific sub-population of Indigenous youth experiences a significant number of stressors and transitions (care placements, transition out of child welfare system etc.), and re-unification with cultural factors could play a significant role in mediating some of the adverse coping mechanisms and behaviors acquired due to forced assimilation into colonization culture (Blackstock & Trocmé, 2005; Ungar, 2006).

## Conclusion

The literature included in this review reported several unique factors and strategies for resilience management in Canadian and American Indigenous youth. By providing flexibility in definitions of resilience, reviewed literature conveyed the complexity of defining, measuring, and predicting Indigenous youth resilience. Despite experiencing a greater amount of stress than settler populations, Indigenous youth have many resilience strategies that they can draw upon to promote holistic health. In supporting Indigenous programming that promotes cultural learning, youth leadership, and relationship building, organizations can promote Indigenous youth and their communities’ resilience.

## Appendix

### Search Terms Used in all Database Searches

(Indigenous*) OR (Indigenous people*) OR (Native*) OR (Native people*) OR (Aboriginal*) OR (Aboriginal people*) OR (First Nation*) OR (Métis*) OR (Metis) OR (Inuit*) OR (Native American*) OR (American Indian*) OR (North American Native*) OR (North American Indian*) OR (Trib*) OR (Band*) OR (Indigenous communit*) OR (Native communit*) OR (Aboriginal communit*) OR (North American Indian communit*) OR (Eskimo*) OR (Alaska* Native*) OR (First People*) OR (Native Hawaiian*) AND (Youth*) OR (Young) OR (Young people*) OR (Young man) OR (Young woman) OR (Young men) OR (Young women) OR (Young girl*) OR (Young boy*) OR (Young female*) OR (Young male*) OR (Adolesc*) OR (Juven*) OR (Teen*) OR (Young Adult*) OR (Child*) OR (Minor*) OR (Pre-pubescent) OR (Prepubescent) OR (Pubescent) OR (Pediatric) OR (Paediatric) OR (Highschool) OR (High school) AND (Resilien*) OR (Adapt*) OR (Positive Adapt*) OR (Persever*) OR (Adjust*) OR (Positiv* Adjust*) OR (Strength*) OR (Mental Strength*) OR (Wellbeing) OR (Well-being) OR (Recover*) OR (Withstand*) OR (Overcom*) OR (Coping) OR (Cope*) OR (Support*) OR (Cultur* intervention*) AND (Canad*) OR (Ontario*) OR (Manitoba) OR (Alberta) OR (Saskatchewan) OR (British Columbia) OR (B.C.) OR (BC) OR (Nunavut) OR (North West Territories) OR (NWT) OR (N.W.T.) OR (Quebec) OR (Newfoundland and Labrador) OR (Newfoundland) OR (Labrador) OR (Prince Edward Island) OR (P.E.I.) OR (Nova Scotia) OR (New Brunswick) OR (United States) OR (US) OR (U.S.) OR (USA) OR (U.S.A.) OR (US of A.) OR (America*) OR (“the States”) OR (Washington) OR (Oregon) OR (California) OR (Arizona) OR (Nevada) OR (Idaho) OR (Montana) OR (Wyoming) OR (Utah) OR (New Mexico) OR (Colorado) OR (Texas) OR (Oklahoma) OR (Kansas) OR (Nebraska) OR (South Dakota) Or (“North Dakota”) OR (Minnesota) OR (Iowa) OR (Missouri) OR (Arkansa) OR (Mississippi) OR (Louisiana) OR (Wisconsin) OR (Illinois) OR (Tennessee) OR (Alabama) OR (Florida) OR (Georgia) OR (South Carolina) OR (“North Carolina”) OR (Virginia) OR (Maryland) Or (Delaware) OR (“New Jersey”) OR (Connecticut) OR (Massachusetts) OR (“New Hampshire”) OR (Maine) OR (Vermont) OR (New York) OR (Pennsylvania) OR (Ohio) OR (Kentucky) OR (Indianna) OR (“West Virginia”) OR (Alaska) OR (Hawaii) OR (“Rhode Island”)
